# Syntactic Iron Foams’ Properties Tailored by Means of Case Hardening via Carburizing or Carbonitriding

**DOI:** 10.3390/ma14164358

**Published:** 2021-08-04

**Authors:** Jörg Weise, Dirk Lehmhus, Jaqueline Sandfuchs, Matthias Steinbacher, Rainer Fechte-Heinen, Matthias Busse

**Affiliations:** 1 Department of Powder Technology, Fraunhofer Institute for Manufacturing Technology and Advanced Materials IFAM, Wiener Strasse 12, 28359 Bremen, Germany; dirk.lehmhus@ifam.fraunhofer.de (D.L.); matthias.busse@ifam.fraunhofer.de (M.B.); 2 Department of Heat Treatment, Leibniz Institute for Materials Engineering IWT, Badgasteiner Str. 3, 28359 Bremen, Germany; ja.sandfuchs@gmx.de (J.S.); steinbacher@iwt-bremen.de (M.S.); fechte@iwt-bremen.de (R.F.-H.); 3 Faculty of Production Engineering, University of Bremen, Bibliothekstraße 1, 28359 Bremen, Germany

**Keywords:** syntactic foam, metal matrix syntactic foam, iron, steel, case hardening, carburizing, carbonitriding, powder metallurgy, metal injection moulding (MIM), vibration damping

## Abstract

Metal foam inserts are known for their high potential for weight and vibration reduction in composite gear wheels. However, most metal foams do not meet the strength requirements mandatory for the transfer of sufficiently high levels of torque by the gears. Syntactic iron and steel foams offer higher strength levels than conventional two-phase metal foams, thus making them optimum candidates for such inserts. The present study investigates to what extent surface hardening treatments commonly applied to gear wheels can improve the mechanical properties of iron-based syntactic foams. Experiments performed thus focus on case hardening treatments based on carburizing and carbonitriding, with subsequent quenching and tempering to achieve surface hardening effects. Production of samples relied on the powder metallurgical metal injection molding (MIM) process. Syntactic iron foams containing 10 wt.% of S60HS hollow glass microspheres were compared to reference materials without such filler. Following heat treatments, the samples’ microstructure was evaluated metallographically; mechanical properties were determined via hardness measurements on reference samples and 4-point bending tests, on both reference and syntactic foam materials. The data obtained show that case hardening can indeed improve the mechanical performance of syntactic iron foams by inducing the formation of a hardened surface layer. Moreover, the investigation indicates that the respective thermo-chemical treatments can be applied to composite gear wheels in exactly the same way as to monolithic ones. In the surface region modified by the treatment, martensitic microstructures were observed, and as consequence, the bending limits of syntactic foam samples were increased by a factor of three.

## 1. Introduction

Metal foams are characterized by low density, high specific stiffness, and superior crash and vibration energy absorption, whereas their strength is reduced in comparison to the respective conventionally produced matrix alloys [1,2,3,4,5,6]. For this reason, metal foams are frequently used in combination with compact materials, e.g., as a permanent insert in cast or molded components, or as the core of sandwich structures [3,7,8]. Typical applications of metal foam structures include crash absorbers for cars and trains, supports for traction batteries of electric vehicles or other lightweight automotive structures, façade elements, roboter handling equipment, and vibration damping supports [9,10,11,12]. In the latter case, the effect of the foam is twofold: first, the foam’s vibration-damping capacity is utilized directly, while secondly, the lower Young’s modulus of the foam leads to a stiffness respectively impedance mismatch at the interface of the foam and the compact component of the composite structure. Elastic waves will thus be reflected at this interface, limiting the transmission of vibrations to other parts of the overall structure. Considering the example of aluminum foam sandwich materials, which are currently used as damping supports of e.g., linear axles, at least two interfaces with impedance mismatch (face sheet to foam and vice versa) have to be transgressed and can thus contribute to vibration alleviation. Advantages of this purely metallic as opposed to an adhesively bonded solution are better long-term stability, a lower risk of aging, and a reduced need for inspection and maintenance.

A further example of metal foams’ general ability to damp and absorb vibration and sound is a prototype study of gear wheels with Foaminal^TM^ aluminum foam insert between gear ring and hub, see Figure S1 (Supplementary Information). In comparison to the original compact steel design, in addition to a weight reduction of 30%, a reduction of gear sound of up to 8 dB in a wide range of rotational speed (300–1000 rpm) and loads (0–100 Nm) was obtained. However, the limited strength of the Foaminal^TM^ foam (density 0.3–0.7 g/cm³, compression strength 2–20 MPa) afforded the addition of a support plate made of a carbon fiber-reinforced polymer (CFRP) to secure sufficiently high levels of transmissible torque [5].

In contrast to conventional two-phase metal foams, of which Foaminal^TM^ is a typical example, syntactic metal foams represent a special subclass of porous metals, as their porosity is not generated e.g., by blowing agents, but instead by the addition of porous or hollow filler particles to the metallic matrix [13,14,15]. Examples of such filler particles are hollow glass microspheres, cenospheres, as well as hollow metal or ceramic spheres [13,16,17,18,19,20]. Syntactic metal foams typically show higher densities than other metal foams like e.g., Foaminal^TM^ or investment cast sponges—specifically, if single-size or at least narrow size distributions of filler particles are used [17]. Due to their higher density, but also due to the intrinsic strength of the integrated hollow particles, the strength of syntactic foams surpasses that of most other types of metal foam [6]. In some cases, they have even been shown to exceed the specific yield strength level of the unfilled matrix material [21]. However, notwithstanding their higher density, they still exhibit the same general features as other foams, including a high deformation and vibration energy absorption capability. Because of this combination of increased strength and considerable damping capacity, metal matrix syntactic foams are natural candidates for application as inserts in gear wheels. In comparison to the aforementioned Foaminal^TM^-CFRP-solution, the possibility to abstain from using an additional reinforcement to realize a purely metallic solution will simplify assembly and processing. It will thus help to reduce costs and avoid foreseeable problems associated e.g., with differences in thermal expansion and thus long-term stability.

For applications focused on weight reduction and improved damping characteristics like the gear wheel example, careful balancing between insert properties, gear and insert geometry, and design loads is mandatory [22,23,24]. Besides their role in vibration attenuation, insert properties and geometries influence the elastic deformation of the loaded gear as a whole. Altogether, too “soft” elastic behavior of the gear can lead to changes of the contact geometries between gear teeth and thus of the conditions during gear engagement. This in turn can cause increased excitation of vibrations and would be counterproductive. Thus, despite the superior damping characteristics of metal matrix syntactic foams, an improper adaption of gear and insert to each other can eventually result in increased vibration and noise levels. Furthermore, changes in actual contact areas of gears need careful consideration in teeth correction not to cause premature failure.

Elastic and plastic behavior of metal foams depends primarily on the properties of the metal matrix alloy and its density; other parameters like pore size distributions are of second-order [2]. Changing the density of a foam from a specific matrix alloy will influence both the elastic and plastic properties in quite a similar way [1,2]. So, for adapting those properties independently, one can revert to heat treatments, like e.g., precipitation hardening in the case of aluminum alloys [25,26]. In this respect, iron and steel matrices are especially interesting as a plurality of hardening mechanisms can be used, including solution, precipitation, and transformation hardening [27,28]. In consideration of the aforementioned application in gears, further advantages are that in the case of the proposed configuration based on a metal matrix syntactic foam insert, the base material of the gear ring insert and hub are similar and can thus be expected to show matching coefficients of thermal expansion and corrosion behavior. Moreover, other than in the case of the steel-Foaminal^TM^ example, the combination provides no driving force for galvanic corrosion and eases recycling.

So far, only very few studies have been published on heat treatment of steel foams in general, and much less covering syntactic iron and steel foams in particular [29,30,31], and all of these focus on hardening rather than thermo-chemical surface-oriented techniques. Taking into account that most high-strength gear wheels today are case hardened, the application of syntactic foam inserts needs to be reviewed regarding actual heat treatment technology of gears to make the same heat treatment of insert and gear possible. The aim of the present work is thus to extend the state of the art by investigating the influence of thermo-chemical heat treatment by case hardening methods, and among these specifically carburizing and carbonitriding—as both types of treatments are frequently used in gear wheel technology—on the structure and properties of syntactic iron foams. Besides shedding light on the associated property change in the foam, the aim is to evaluate the general response of these materials to the treatment, and thus to establish whether a compound design incorporating both conventional steel and steel matrix syntactic foams can be subjected to these processes without compromising foam structure.

The study included two-sphere content levels (reference 0% and 40%) and three heat treatment conditions (as-sintered, carburized, and carbonitrided). The sphere content was not varied further as, (a) for much lower sphere contents both the decoupling and the damping capacity of the foam insert would be reduced significantly, and (b) at much higher sphere contents the reduced strength and ductility of the syntactic metal foams might be insufficient for the target application (e.g., [4,15]).

## 2. Materials and Methods

### 2.1. Production of Foam Specimens

The production of the syntactic iron foam samples for the subsequent hardening treatments followed the approach described in [18,32], i.e., hollow glass microspheres were used as an additive to the feedstock processed in conventional metal powder injection molding (MIM). The MIM process consists of the following steps: feedstock preparation, injection, chemical debinding, thermal debinding, and sintering, see Figure S2.

The primary materials for the experiments were iron powder of grade Diafe 5100 (Dr. Fritsch, Fellbach, Germany) and micro hollow glass spheres S60HS (producer 3M, supplied by Omya, Cologne, Germany). The characteristics of both materials are listed in Table 1. With Diafe 5100, a rather fine iron powder with high sintering activity was chosen to account for the low softening point of the S60HS hollow glass sphere, which limits the allowable sintering temperatures [33,34].

Two feedstock batches of 400 mL each were produced. For the first batch (reference material), 60 vol.% of iron powder and 40 vol.% of binder (mixture containing paraffin, lupolen, and stearin) were processed for 2 h at 120 °C in a mixer (self-constructed equipment based on Brabender components), cooled to room temperature and granulated. The second batch (syntactic foam) was processed in a similar way, however, after 90 min of mixing time the S60HS spheres were added to the feedstock. The mass fractions of the mixtures are noted in Table 2. The added mass of the S60HS spheres was calculated in order to adjust the sphere volume content to 40%. This correlates with the following theoretical volume contents: metal 60%, glass 11%, and void 29%. The composition was chosen this way because it was known from earlier investigations that syntactic metal foams with 40 vol% spheres exhibit a good compromise of remanent strength and increased damping resp. decoupling [4,21].

The injection molding was performed on a BOY XS type injection molding machine (Dr. BOY GmbH), Figure S3. The dimensions of the rectangular mold cavity were 40 mm × 15 mm × 5 mm. Molding parameters are summarized in Table 3.

After injection, the samples were examined using radioscopy (MU2000, 160 kV, YXLON International GmbH, Hamburg, Germany) and debinded chemically (model series EDA, LÖMI GmbH, Aschaffenburg, Germany) for 24 h in hexane at 35 °C, then dried for 16 h and subsequently transferred to the sintering furnace (Thermal Technology LLC, Santa Rosa, CA, USA, support: alumina plates). Thermal debinding and sintering took place in a hydrogen atmosphere. The temperature program is depicted in Figure S4. The initial heating rate from room temperature to 100 °C was 2 K/min, between 100 °C and 500 °C a rate of 0.1 K/min was used. For thermal debinding a holding step at 500 °C was inserted. Afterwards, the heating rate was increased to 3 K/min. The sintering itself is done at 900 °C for 3 h. The cooling after sintering was relatively slow (appr. 2 K/min down to 300 °C, then 1 K/min). After sintering, the samples were checked once more by means of radioscopy (YXLON MU2000, 160 kV). Sample dimensions were determined manually and shrinkage was calculated from the differences observed between green and sintered parts dimensions. The density of samples was measured by means of Archimedes’ method.

### 2.2. Heat Treatment, Hardening

**Figure 1:**
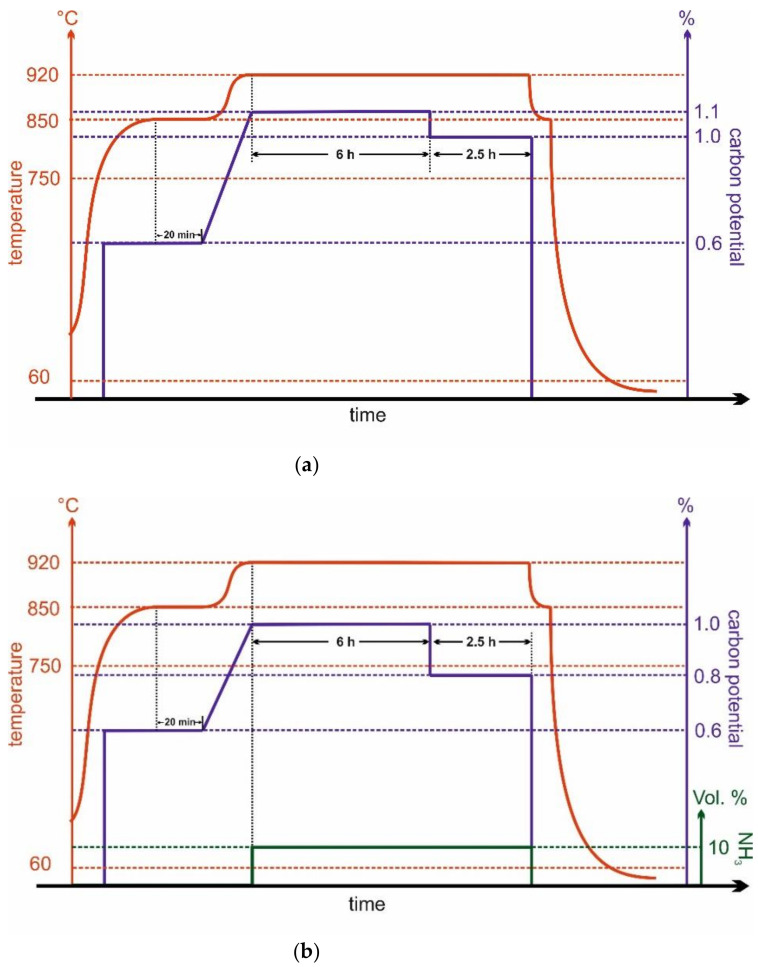
(**a**) Schematic T-t-C-level-course of the carburizing during the case hardening process, and (**b**) the parameters of the carbonitriding process.

The case hardening treatment was conducted using a bell furnace (type 11CG, SOLO Swiss SA, Porrentruy, Switzerland, Figure S5) with a nitrogen–methanol atmosphere. The carbon level was automatically controlled by a control system (Process-Electronic GmbH/United Process Controls, Heiningen, Germany) using an oxygen probe. All gaseous and liquid media was introduced through liquid flow (LFC) and mass flow controllers (MFC). The carburization was performed according to the time-temperature program shown in Figure 1a. Samples were prepared on a steel batch tray in a hanging positon.

The batch was introduced into the hot furnace (850 °C), directly heated to 850 °C, and held there for 30 min to achieve a temperature homogenization. Carburizing started at 750 °C with a carbon level of 0.6%. Thereafter, heating to carburizing temperature was started until 920 °C was reached. The main carburizing step took place at 920 °C and C-levels of 1.1% respective 1% for carburization. Afterwards, the specimens were quenched directly from 920 °C in water at RT and tempered for 1 h at 180 °C.

The program for the carbonitriding treatment of the samples closely followed the carburizing with only slightly adapted carbon levels and the addition of 10 vol.% ammonia, see Figure 1b. The C-level was slightly reduced to achieve a sufficient solubility for both carbon and nitrogen. After carbonitriding, the specimens were quenched in water at RT and tempered for 1 h at 180 °C.

### 2.3. Metallographical Investigations and Mechanical Testing

After the case-hardening by carburizing respective carbonitriding of both reference and foam samples, one sample per batch was used for metallographic investigations. Samples were polished and subsequently etched 20–25 s using 3% HNO_3_ (Nital). On the reference samples, Vickers hardness (HV1) measurements were performed in order to determine hardness profiles normal to the sample surface. Similar measurements were not feasible in the case of the syntactic foam samples, as the diagonal length of the Vickers indentations is in the same order of magnitude as both the diameter of the hollow microspheres integrated into the metal matrix and their average distance. Therefore, any indentation will necessarily be positioned close to several microspheres, which leads to a large scatter of the observed hardness values and prevents the establishment of reliable hardness values and local material condition, i.e., heat treatment state.

Sparc optical emission spectrometry (S-OES/ARL^TM^ 3460 Thermo Fisher Scientific, Dreieich, Germany) was again applied only to the reference samples. Previous experiments with foam samples had shown that measurements on syntactic foams lead to large scatter and unrealistic compositional information. Five measurements per sample and depth were carried out; the focal spot diameter of the measurements was 6 mm with a depth of 10–30 µm. Before measurement, the sample surfaces were leveled slightly using a zirconia grinding wheel. For each depth of the profile, grinding by the zirconia wheel was performed with repeated measurements of local carbon content at the specific depth. Thus, a discrete point-to-point carbon profile was derived by this method. To maintain the accuracy of the measured carbon contents multiple standard samples were used to calibrate the machine data.

Using the hollow sphere-free sample was mandatory to achieve any process control by the means of carbon profiling. Obviously, some deviations are to be expected on the carbon profile of the samples with spheres because the additional surface tends to speed up diffusion in the material.

For the determination of mechanical properties, 4-point-bending tests were performed. Bending test samples were produced from sintered samples by milling and burring, final cross-section dimensions were 7 mm × 3.7 mm, length was in the range of 33.5–34 mm. Both for reference and syntactic foam materials, batches of seven samples each were carburized respective carbonitrided and hardened in water. To prevent a thorough carburization, the flanks of the samples were protected against carburization using the carburization protection paste Condursal 777 (NÜSSLE GmbH & Co.KG, Nagold, Germany) on both sides of the sample (7 × 40 mm flanks). Twelve samples—six with and six without microspheres—remained untreated.

For the 4-point-bending test, two samples were equipped with strain gauges, with data monitoring conducted via a quarter bridge circuit, a multiplier of type Picas (Peekel Instruments, Gemsbach, Germany), and the WaveMatrix (Instron GmbH, Darmstadt, Germany) software used for data analysis. Calculation of the bending stress σ_B_ was based on the formula

(1) $$\sigma_{B}=\frac{3F\left(L-l\right)}{2bh^{2}}$$

with *F* the applied force, *L* the support span, *l* the loading span, *b* and *h* the width and height of the sample. Nominal sample dimensions have been given above, for the actual experiments, and their evaluation, width, and height were measured individually on each sample. The length of the support span was 28 mm, those of the loading span were 11.5 mm. The experimental setup is shown in Figure S6. Not all samples were evaluated using strain gauges to achieve data on effective strain rates during testing. Mostly for the case hardened samples the effective ductility was that low, that an evaluation of the straining could be derived from elastic deformation only by using the displacement sensor of the machine. The bending modulus was determined from the slope of a linear regression line in the quasi-elastic initial region of the bending stress-deflection diagrams (ranging from test start to the bending limit).

## 3. Results

Radioscopy showed that both the as-injected green parts, with and without hollow sphere additions, were mostly flawless. The subsequent chemical debinding and sintering did not lead to crack formation, except for a very few cases. For the subsequent tests, only specimens without cracks or exceptional porosity were used. The densities of the sintered specimens of the different batches are summarized in Table 4 and compared to the theoretical respective literature values. The theoretical density of the syntactic iron foam was calculated according to the formula:

(2) $$\rho_{f,th}=\langle 1-\phi_{sph}\rangle *\rho_{fe}+\phi_{sph}*\rho_{sph,tr}$$

with *Φ_sph_*, *ρ_sph,tr_* the volume content respective the true density of the hollow glass spheres and *ρ_fe_* the density of the iron matrix (7.787 g/cm^3^). An additional comparison is based on the reference density of pressed and sintered iron, according to [34]. The linear shrinkage in three dimensions is listed in Table 5.

**Figure 2:**
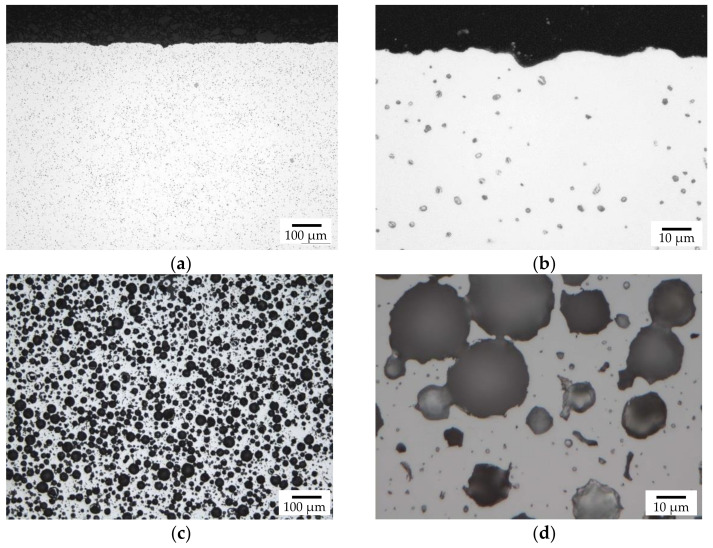
Macroscopic overview of the polished section of an as-sintered sample without hollow, (**a**,**b**) and with spheres (**c**,**d**).

The results of the metallographic investigation of the un-treated, as-sintered samples are shown in Figure 2. As was expected from the density measurements, the metal matrix contains some residual sintering porosity, uniformly distributed over the area of the metallographic section. The size of these pores is in the range of 2–6 μm. Most of the hollow glass spheres are intact, several are deformed, and a significant number of neighboring spheres appear to have merged, while others have melted. The results of the S-OES (optical emission spectroscopy) measurements are listed in Table 6.

**Figure 3:**
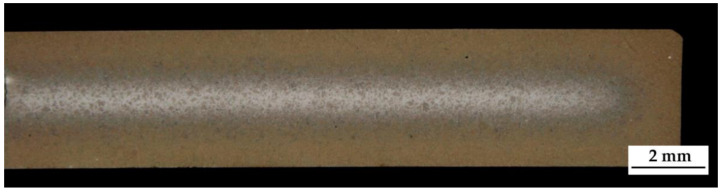
Macroscopic overview of the polished section of the carburized sample without hollow spheres. Color differences provide a visual idea of the hardening depth.

**Figure 4:**
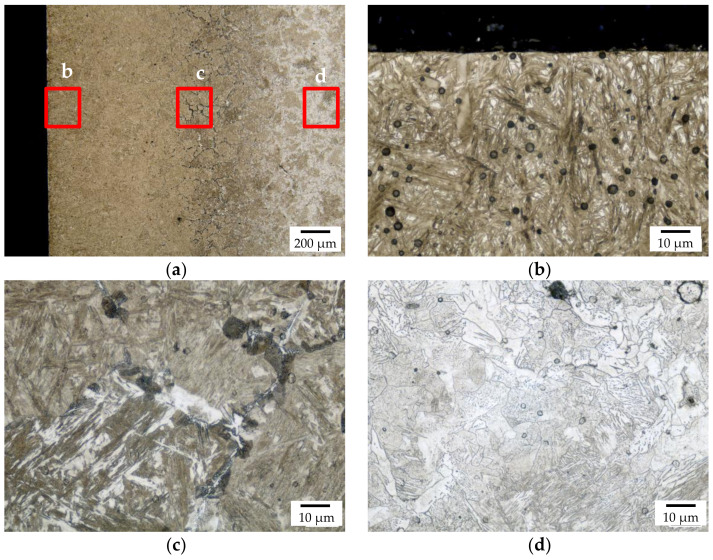
Detailed view of the polished section of the carburized sample without hollow spheres with (**a**) showing the overview of the case, (**b**) showing the surface near layer, (**c**) showing the transition zone at the case hardening depth CHD, and (**d**) showing the core microstructure.

The macroscopic metallographic sections of the carburized samples without hollow spheres featured clearly distinguishable core, transition, and carburized areas (Figure 3). The case reaches deep into the samples leaving only little material which was non-carburized. Detailed views of the different areas are shown in Figure 4. The case area is characterized by a mainly martensitic structure with finely dispersed retained austenite. In comparison, the cores exhibit martensite, bainite, and perlite. Furthermore, small residual sintering pores can be observed in the iron matrix. Because of the non-ferritic core, it must be assumed that the carbon profile reaches down into the core of the sample.

**Figure 5:**
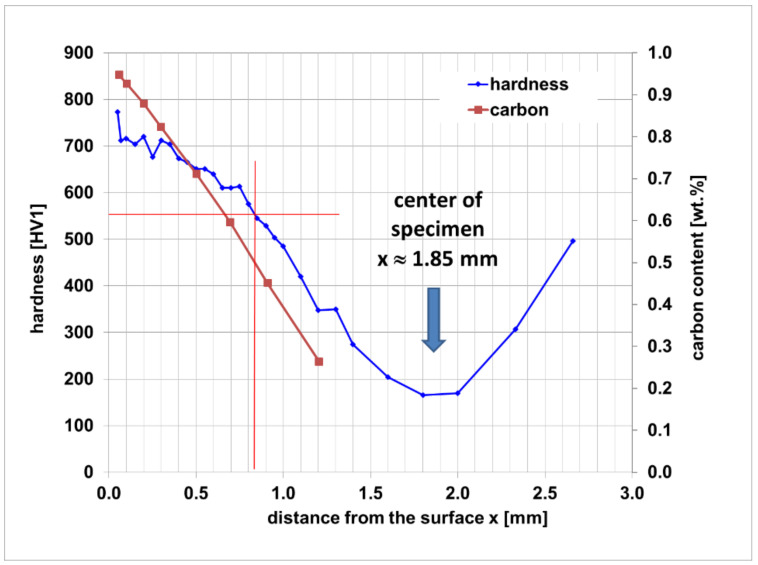
Hardness (HV1) and carbon content profile of the carburized sample without hollow spheres.

The Vickers hardness and carbon content profiles are shown in Figure 5. Hardness measurements were performed “case-to-core” of the sample since the metallographic section in Figure 3 shows that the microstructure of the sample is symmetrical with no indication of any dependency of the hardness profile on the sample face. The maximum hardness is 720 HV1 at a distance of 0.2 mm from the sample surface. The minimum hardness is 166 HV1 in the center axis of the sample. The case hardening depth (CHD) according to standard DIN 50190-1 [35] was determined to be 0.84 mm.

**Figure 6:**
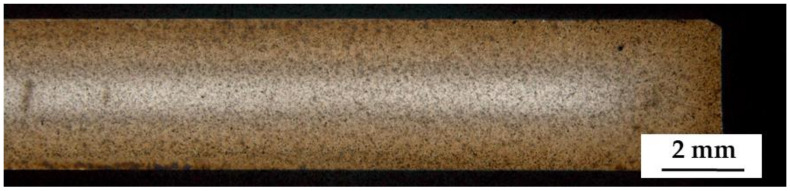
Macroscopic overview of the polished section of the carburized sample with hollow spheres.

**Figure 7:**
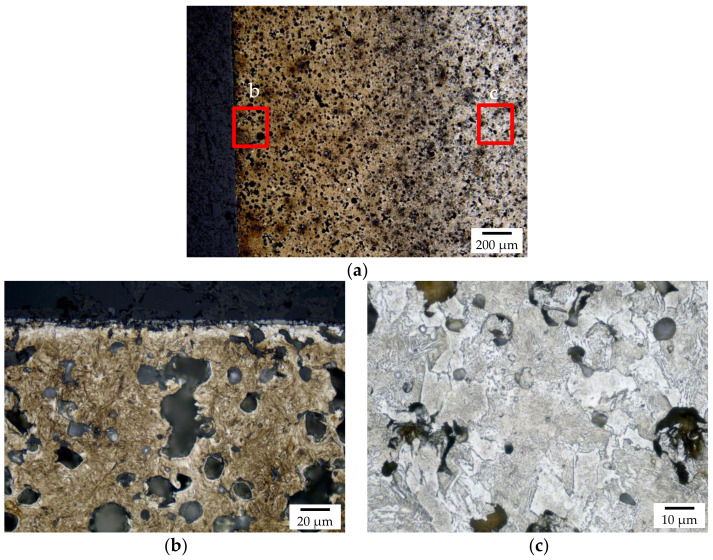
Detailed images of the polished section of the carburized and hardened sample with hollow spheres with (**a**) showing the overview of the case, (**b**) showing the surface near layer, and (**c**) showing the core microstructure.

Macroscopic metallographic sections of the carburized samples with hollow spheres are displayed in Figure 6 and Figure 7. As for the sample without hollow spheres, core, case, and a transition zone can clearly be distinguished. The case is again characterized by a martensitic structure with finely dispersed retained austenite; the core contains bainite and perlite, with martensite missing in this region, and in contrast to the reference compact samples. The presence of a non-ferritic microstructure in the core of the sample means that some carbon has reached the core of the sample at a low magnitude. On the surface of the sample, a thin oxide scale can be observed being formed during the transfer of the samples from the furnace into the quenching tank through the air. Again, most of the hollow glass spheres are intact, but several are deformed, and a significant number of neighboring spheres appear to have merged, while others have melted. This is somewhat surprising given that the heat treatment temperature exceeds the sintering temperature by only 20 °C.

**Figure 8:**
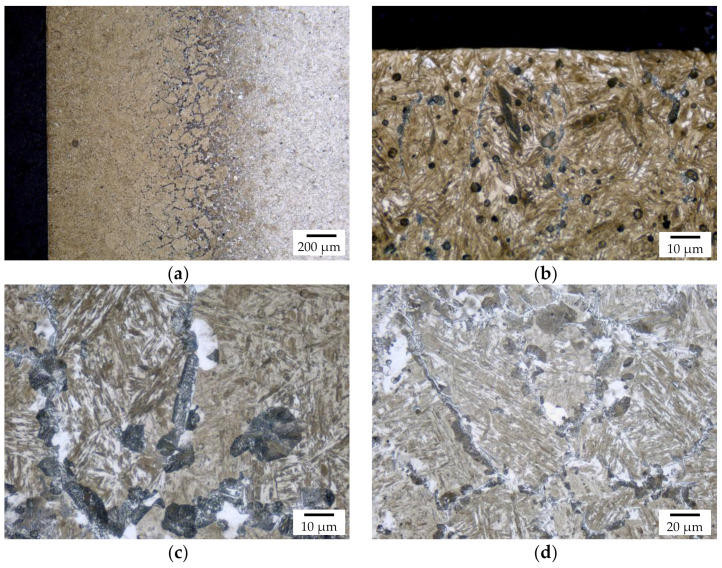
Detailed images of the polished section of the carbonitrided sample without hollow spheres; (**a**) location of the respective position relative to the core, case, and transition zone, (**b**) view of the case, (**c**) transition zone, and (**d**) core.

**Figure 9:**
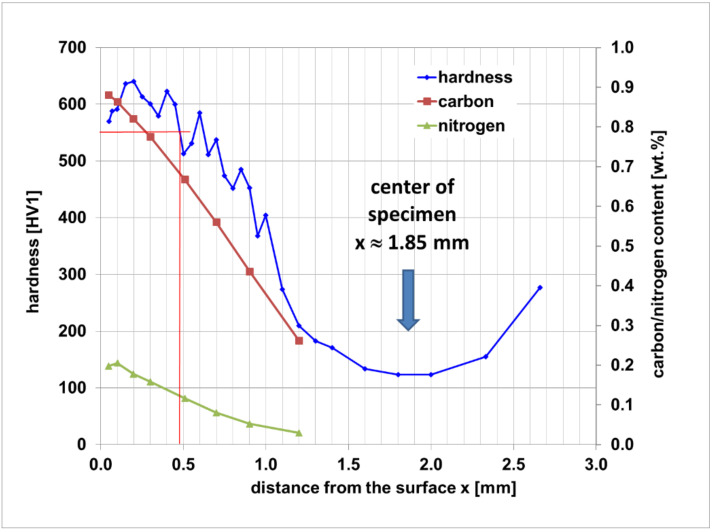
Hardness (HV1), carbon, and nitrogen content profile of a carbonitrided sample without hollow spheres.

The macroscopic structures of the carbonitrided samples without and with hollow glass spheres are similar to the respective carburized ones; in each batch core, case, and transition zone can clearly be identified. For samples without hollow spheres, the case exhibits martensite with finely dispersed retained austenite and some low amounts of troostit being visible on the former austenite grain boundaries; the core shows martensite, bainite, and perlite, Figure 8. The main structural components are thus similar to the batch of carburized samples without hollow spheres. The HV1 hardness profile is shown in Figure 9. Again, hardness measurements were performed “case-to-core” of the sample. The hardness maximum is detected at a distance of 0.2 mm from the surface at 640 HV1. The hardness remains on a level of 600 HV1 up to a distance from the surface of 0.4 mm and decreases to a minimum of 124 HV1 in the samples’ center. From the data, a hardening depth of 0.48 mm was determined following the standard procedure laid down in DIN 50190-1.

The carbon and nitrogen content profiles, determined by S-OES, are also shown in Figure 9. The surface carbon content (at 0.05 mm distance from the surface) is 0.88 wt.% and decreases almost linearly with increasing distance from the surface to a level of 0.262 wt.% at 1.2 mm depth. The carburizing depth is 1.06 mm. The surface nitrogen content is 0.198 wt.% at a depth of 0.05 mm. The nitrogen content increases to a maximum value of 0.206 wt.% at 0.1 mm and decreases then also almost linearly to a level of 0.029 wt.% at a 1.2 mm distance from the surface.

**Figure 10:**
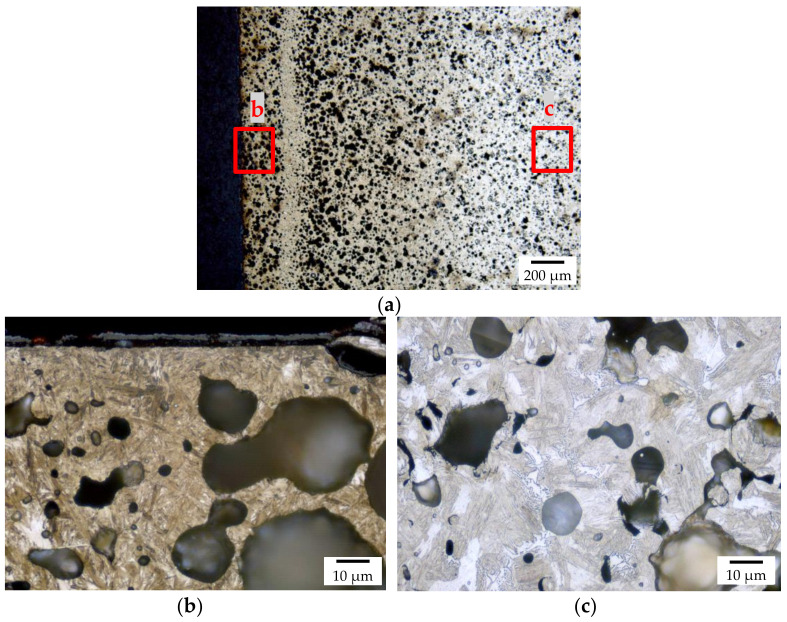
Detailed images of the polished section of the carbonitrided sample with hollow spheres with (**a**) showing the overview of the case, (**b**) showing the surface near layer, and (**c**) showing the core microstructure.

Detailed images of the metallographic section of carbonitrided samples with hollow spheres are shown in Figure 10. The surface near the carburized layer contains (as in the case of all other batches) martensite and fine retained austenite, and the core contains some martensite and mainly bainite. Again, the hollow spheres are generally in good condition, but significantly deformed with a fused appearance; often, neighboring spheres seem to have merged.

**Figure 11:**
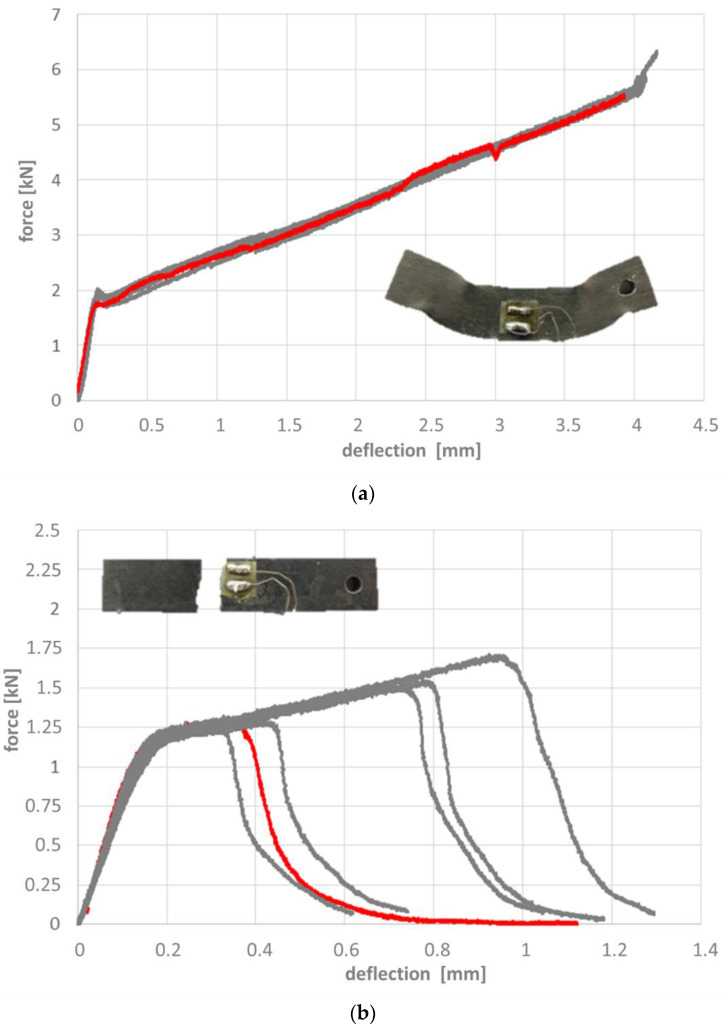
Force-deflection curve for samples without heat treatment, (**a**) without, (**b**) with hollow glass microspheres. Inserts show the samples corresponding to the red curve. Note the difference in scale of both axes.

**Figure 12:**
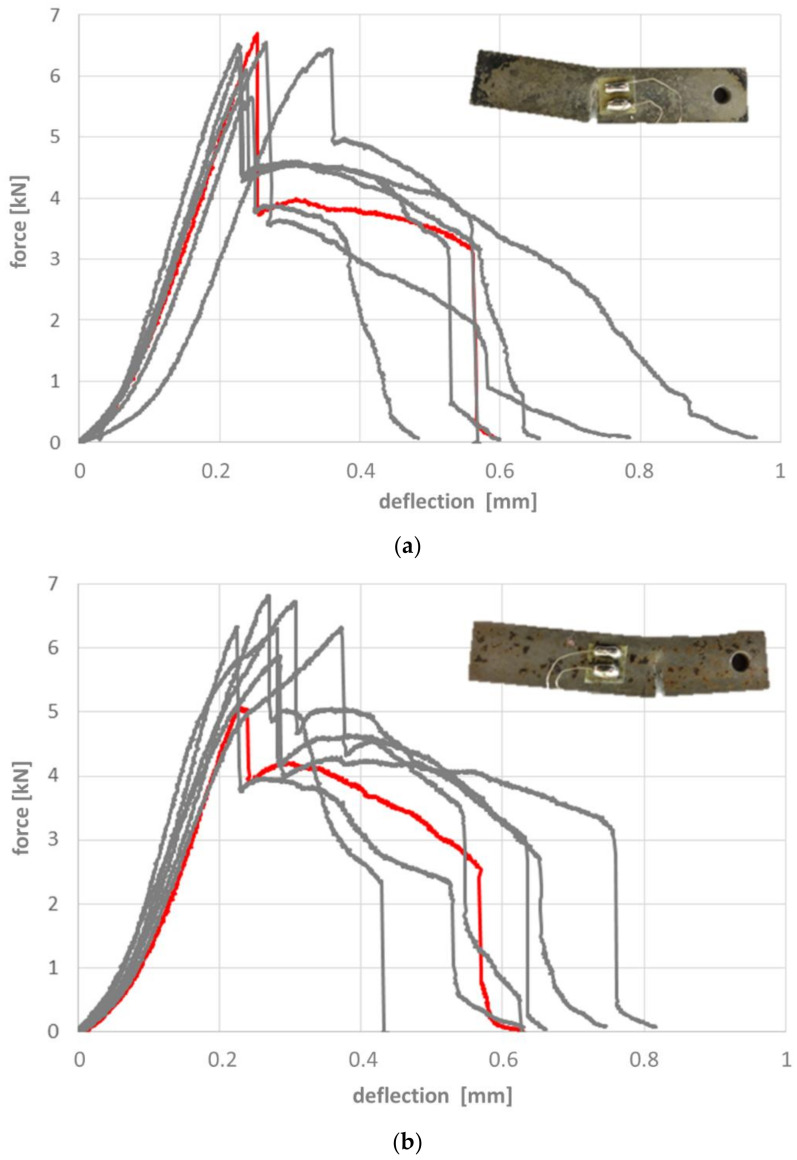
Force-deflection curves for samples without hollow glass microspheres, (**a**) carburized, and (**b**) carbonitrided. Inserts show the samples corresponding to the red curve. Note the difference in scale of both axes.

**Figure 13:**
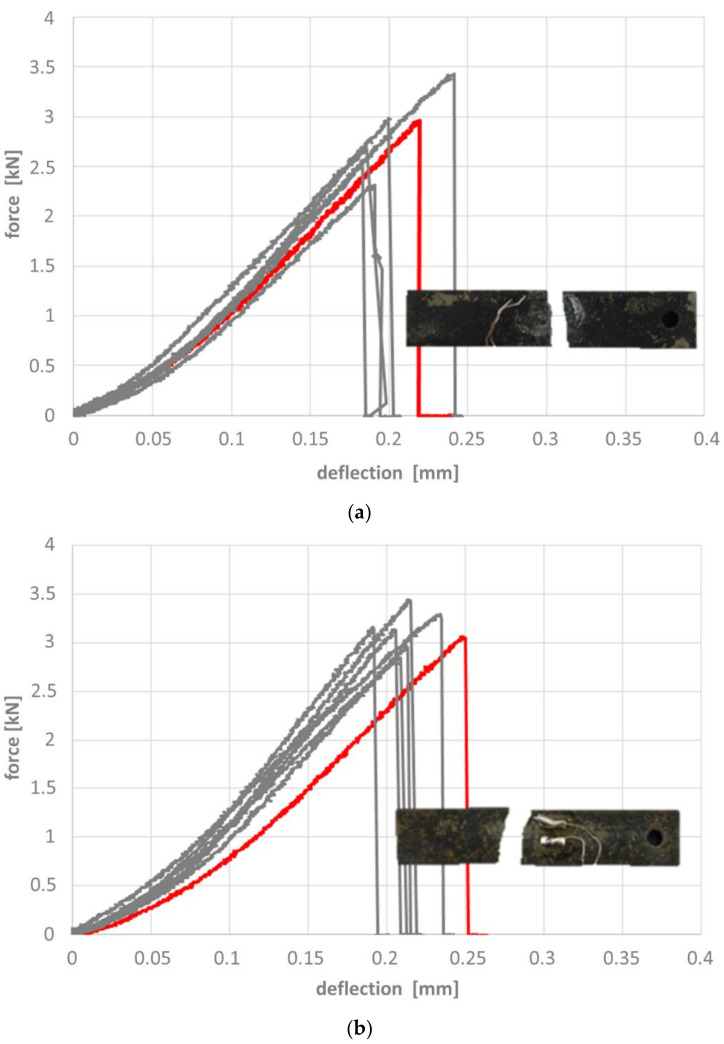
Force-deflection curves for samples with hollow glass microspheres, (**a**) carburized, and (**b**) carbonitrided. Inserts show the samples corresponding to the red curves.

Figure 11, Figure 12 and Figure 13 depict the results of the 4-point-bending tests based on the force-deflection curves recorded. Representative samples in their post-testing condition are shown as inserts in these figures. The respective curves can be associated with four fundamentally different types of failure, which correspond to samples without heat treatment, without and with microspheres (Figure 11a,b), samples with heat treatment without microspheres (Figure 12), and samples with heat treatment and microspheres (Figure 13). In other words, this means that the way in which the failure mode of reference samples and syntactic foam samples is influenced by the heat treatment is independent of the type of heat treatment, i.e., a case-hardened reference sample will deviate in basically the same way from a reference sample without heat treatment as a carbonitrided one. The same is true for syntactic foam samples. However, the failure modes of heat-treated reference samples differ from those of the heat-treated syntactic foam samples, just as the sintered reference samples differ from the syntactic foam ones.

Un-treated, as-sintered iron samples without filler failed in a predominantly ductile manner. The force-deflection curve pictured in Figure 11a is characterized by a constant increase following the initial elastic region. This behavior can be explained by the specific deformation pattern of this type of sample as exemplified in the insert in the aforementioned Figure 11a. The reference samples’ high level of ductility causes the region of plastic deformation to move from the area between two load introduction points to the region between load introduction and support points.

To a limited extent, this behavior is mirrored by the force-deflection curves measured on syntactic foam samples without heat treatment, which also show plastic deformation, but no localization of the same. Instead, these samples fracture at varying levels of deformation (Figure 11b).

All non-heat treated samples show a distinct mixed-mode elastic-plastic deformation from the beginning of loading, making a distinction of a clearly elastic deformation very difficult. Therefore the localized deformation changing over from elastic-plastic bending with a characteristic bending behavior, equally distributed between the two-force inducing points, to a localized deformation at the load-inducing points is taken as a marker for stating the failure of the sample.

Carburized and carbonitrided unfilled samples respond to the bending test in altogether three stages: the elastic region is followed by the initiation of a crack, which however does not transgress the full sample, but is apparently arrested in its more ductile core. This results in a steadily declining shape of the force-deflection curve which is in some cases interrupted by a second fracture initiation. The scatter in terms of deflection and thus strain at the first occurrence of a crack observed for carburized and carbonitrided samples is larger in the latter case (Figure 12).

In contrast to the unfilled samples with heat treatment, the corresponding syntactic foam samples fail in a completely brittle manner. Once a certain stress level is reached and a crack is created, the latter will immediately pass through the whole sample. Both force and deflection are slightly higher at this point in the case of the carbonitrided samples, which also show slightly less scatter of the parameters in question than their carburized counterparts (Figure 13).

**Figure 14:**
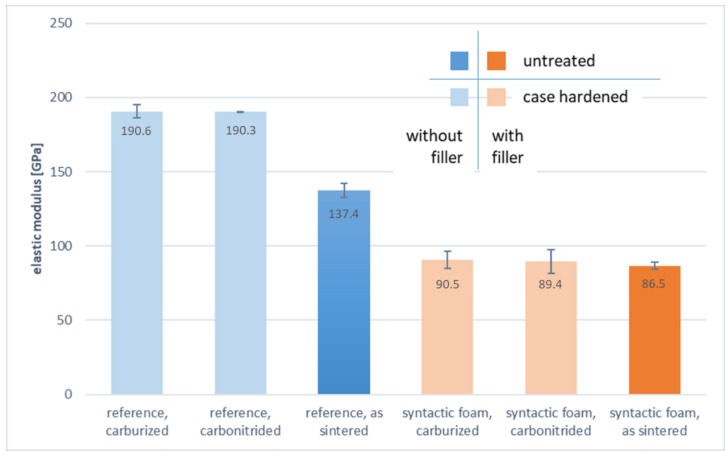
Comparison of the approximate elastic (bending) modulus of the various types of samples with/without heat treatment and with/without filler (with standard deviation).

Interestingly, the lower elastic modulus of the foam samples allows them to reach the same level of deformation at failure as the associated reference samples despite the formers’ lower strength. The moduli were determined by means of linear regression of stress-strain curves derived from the force-deflection data using an equation to determine stress as well as the readings from the strain gauges; the material and condition-dependent values obtained are collected in Figure 14.

Not surprisingly, and in line with general notions on the characteristics of porous materials, the comparison of the elastic modulus values summarized in Figure 14 reveals a reduction of this property which is clearly associated with the addition of filler particles; whereas samples without these reach levels between 130 and 190 GPa, and syntactic foams remain in a range of 80 to 100 GPa. Looking at the reference samples, it is noteworthy that heat treatment results in an apparent increase of the bending modulus when opposed to specimens in the as-sintered state; this effect is missing in the case of the syntactic foam samples, which all show identical values of the elastic modulus with slightly higher scatter if relative rather than absolute figures are considered. This observation can be seen as an indication that for the reference samples without heat treatment, some plastic deformation is occurring already within the deformation range taken as the basis for the determination of the modulus values, effectively making this an elasto-plastic rather than a truly elastic range.

**Figure 15:**
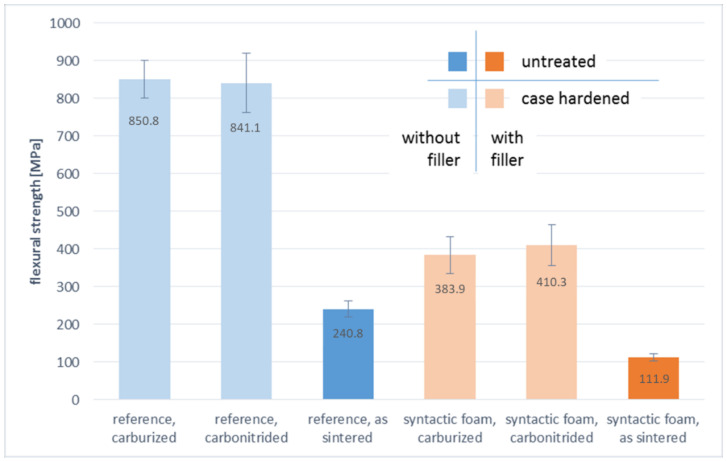
Bending limit of the different material batches (with standard deviation).

The bending limits of the different batches are contrasted in Figure 15. Data scattering is comparatively low. The diagrams show that carburizing and carbonitriding lead to a significant increase of bending limit both for the reference material and the foam. However, no significant difference between the two treatments can be found, the bending levels are quite similar in each case. This reflects the earlier observation that within each of the two groups, the failure mode does not change with the heat treatment either.

Altogether, the bending limit of the carburized and carbonitrided syntactic foams is significantly lower than those of their non-porous counterparts but higher than that of the as-sintered reference samples. The factor of approximately 3.5, by which both heat treatments increase the bending limit, is similar for reference and foam samples.

While the differences in the heat treatments effects is insignificant in the case of the reference samples, the effect of carbonitriding appears to exceed that of carburizing for the syntactic foam samples, although the hardness is lower.

## 4. Discussion

The lower density of the sintered iron samples without and with hollow spheres in comparison to the reference densities of pure iron and the theoretical foam density can be explained by the use of MIM and relatively low sintering temperatures. Hollow glass spheres like S60HS are not suitable for PM-processes like uniaxial pressing because of their limited crush strength. Furthermore, the large difference in density between hollow spheres and iron particles results in a pronounced demixing tendency. Using injection molding, those problems can be circumvented. A large amount of binder of the feedstock effectively prevents demixing and the moderate shaping temperatures and pressures ensure the survival of the hollow spheres. On the other hand, the metal powder bulk of MIM parts in the initial sintering phase resembles that of a loose powder, and a relatively large densification has to be obtained by the sintering. The relatively moderate sintering temperature (900 °C) is necessitated by the limited thermal stability of the S60HS spheres. Though it is known that such spheres, embedded in fine metal powder, can survive temperatures much higher than their softening point of 600 °C, it was shown in earlier investigations that low process temperatures and fine, highly sintering-active metal powders are preferable to retain the integrity of the microspheres [36]. On the other hand, one also has to consider the higher costs for very fine metal powder. In comparison to earlier works somewhat coarser iron powder was used in the present investigation—d_50_ = 2.5 μm in comparison to d_50_ = 1.4 μm in [37]. The sintering density of the compact specimens is comparable to the results of Tavares et al. [38]. The relative deviation of theoretical and measured densities is somewhat smaller for the foam than for the compact samples. This can be attributed to the fact that during softening and deformation hollow spheres can exhibit volume shrinkage. The residual porosity of the metal matrix is, therefore, balanced to some extend by shrinking hollow spheres [39]. The shrinkage of the samples is isotropic, indicating neither gravity-induced deformation nor collapse of the soft hollow spheres during sintering. The related volume shrinkage (0.85 × 0.85 × 0.85) matches the binder volume content and the observed residual porosity.

The sintering pores remaining in the metal matrix are small and globular for both the compact as the foam samples. In the case of the foam samples, the influence of those pores on the overall mechanical behavior should be negligible.

Because of the analytical restrictions caused by the hollow spheres embedded in the metal matrix, a direct comparison of reference and foam samples was only possible by means of optical microscopy of the respective metallographic sections, and by mechanical characterization. Based on the observed, clearly expressed case with a mainly martensitic microstructure, and mostly bainite and some martensite in the samples’ cores, it can be concluded that clearly both compact and porous samples can successfully be case-hardened by carburization or carbonitriding with subsequent water quenching. Water quenching was necessary because carbon and nitrogen were the only hardenability-increasing elements in the metal powder used. The core hardness of the case hardened compact samples was, at 124 HV1 and 165 HV1, respectively, more than doubled in comparison to pure ferritic iron (60 HV1 [40]).

The only significant structural differences in samples with deviating heat treatments are found in the core of foam samples hardened using carburizing and carbonitriding: the former exhibits bainite and perlite, the latter martensite, and bainite. This concurs with the fact that enrichment of nitrogen and carbon in the iron matrix reduces the critical cooling rate for the hardening and the martensitic transformation [41].

It can be assumed that the superficial heat transfer conditions during quenching are similar for all samples, as MIM-based syntactic foam samples tend to show no microspheres on the surface. The internal thermal situation is quite different, however, for the compact and porous samples. Introducing the hollow glass spheres into the metal matrix changes both the heat capacity (respective thermal energy to be transported) and the thermal conductivity of the samples. Neither porosity nor glass walls will contribute appreciably to the thermal conductivity (the thermal conductivity of borosilicate glass is 1.2 W/m/K in comparison to 40–60 W/m/K for low alloyed steel). Furthermore, the intrinsic structure of foams leads to longer thermal diffusion paths. According to [2], the relation

(3) $$\lambda_{f}=\lambda_{m}{\left(\frac{\rho_{f}}{\rho_{m}}\right)}^{q}\;with\;1.65<q<1.8$$

can be used for estimating the foam’s thermal conductivity, with *λ_f_*, *λ_m_*, *ρ_f_* and *ρ_m_*, the thermal conductivities and the densities of the foam and the matrix, respectively. When only the metal content (95.1 wt.%) is taken into account in the foam, Formula (2) shows that this property is reduced to approximately 0.36–0.39 *λ_m_* in the foam. In comparison, the heat capacity (to which both iron and glass contribute) is 0.63 of the heat capacity of the compact iron. As a result, the cooling rate of the foam is lower than that of the compact material, concurring with the fact that martensite formation is partly suppressed and perlite retained in the core area of the carburized foam. 

In the investigation of the carbon and nitrogen profile derived from carbonitriding or carburizing, carburizing and carbonitriding effects were in good agreement with the expected behavior of a metal sample made of unalloyed steel. Taking into account the characteristics of the iron base of the powder used for feedstock production of both the MIM syntactic foam and the reference samples made without spheres, the carburization led to a surface carbon content in the range of the applied carbon level. The profile of carbon in both processes is mostly linear decreasing from surface to core because of the carbon level being held at one stage during the entire process. By carbonitriding, the surface carbon content is lower (about 0.1 wt.%) than in the carburized sample which is in agreement with the effective c-level being set to 0.8% during the second stage of carbonitriding. By the addition of 10 vol.% ammonia to the atmosphere, a surface nitrogen content of about 0.2 wt.% was achieved. This is again in good agreement with similar investigations where unalloyed steels tend to achieve a nitrogen content of no more than 0.2 wt.% at the given ammonia addition and temperature.

The depth of the carbon penetration was in both processes that deep, that the core material is showing some light carburization making the core hardenable by martensitic and bainitic transformation. Without the addition of carbon during the carburization in the core, a fully ferritic matrix would have been achieved which is from a strength perspective not favorable for the bending beams. Although the material with and without spheres exhibits some porosity after sintering, no abnormally fast diffusion of both carbon and nitrogen could be observed in the profiles. This points to a good material density achieved by sintering, leaving no surface-active diffusion component being driver for extremely high diffusion rates. Therefore carburization is comparable to a steel sample made of a steel bar from a conventional steel route.

The hardening of the samples being carbonitrided and carburized was performed in water at RT. Water was chosen to achieve a good hardenability and a sufficient hardening depth in the unalloyed steel matrix. The results indicate that for both processes, a full martensitic case could be achieved with a core microstructure composed of mainly bainite and martensite. The slightly increased core carbon content helped to achieve some hardness increase. The surface carbon content seems to be a major driver in this unalloyed steel to achieve sufficient hardness. For the tempered sample having been carburized, a surface hardness of about 700 HV1 was achieved which is in very good agreement with a case hardened sample made of conventional steel. The carbonitrided sample showed a slightly lower hardness of up to 650 HV1 at the surface, having a 0.1 wt.% lower surface carbon content being the driver for the changed hardness. The additional nitrogen content of about 0.2 wt.% obviously could not compensate for the lack of 0.1 wt.% carbon.

The surface microstructure for both the carburized and the carbonitrided sample is mostly martensite with some retained austenite. The carburized sample with a slightly higher surface carbon content showed a little more retained austenite in the metallographic cuts than the carbonitrided sample. This is in good agreement with the fact that carbon is lowering the Martensite-Start (Ms) and Martensite-Finish-temperature (Mf) twice as much as nitrogen, but nonetheless surprising as nitrogen and carbon in the carbonitrided sample should in sum achieve the same Ms and Mf temperature as in the carburized-only sample. Taking the results achieved by the samples into account the effective carbon profile should have been the same for both heat treatment variants adding only a nitrogen profile by the carbonitriding process to achieve a little increase in retained austenite content.

Though the hollow spheres have deformed due to the high process temperatures of sintering and case hardening, no indication could be found for sphere destruction by the reaction of SiO_2_ (the main component of borosilicate glass) with the diffusing carbon or nitrogen. This could be expected, as the enthalpy of the formation of CO is more negative than that for SiO_2_ only for temperatures above 1500 °C.

The effects of the case hardening are clearly reflected in the results of the bending tests. As already described above, altogether four fundamentally different responses to these experiments could be distinguished, all of them clearly linked to (a) presence of microspheres and (b) heat treatment state of the specimens. These are summarized in Table 7 below.

The presence of an initial elastic or elasto-plastic region prior to any localization of deformation allowed determination or, in the case of the syntactic foams, approximation of Young’s modulus using data from the strain gauges mounted on the samples between the load introduction points (data summarized in Figure 14). In the comparison of the respective data, the reduced elastic modulus of the samples containing hollow glass microspheres obvious, which is typical for foams in general, and thus also for syntactic foams [42], and which enters as a linear factor into the formula for flexural rigidity of the samples. As expected, the case hardening treatments did not have a significant influence on the slope of the curve in this (quasi-elastic) bending regime.

Looking at the data on bending limits as depicted in Figure 15, it is noteworthy that when comparing reference and syntactic foam samples, the latter maintains an advantage corresponding to a factor of approximately 2.2 over their counterparts in both untreated and the two case hardened conditions. However, because the case hardening treatment obviously has a significant effect on matrix properties, as is also proven by the hardness profiles obtained for the reference samples, the heat-treated syntactic foam samples outperform the as-sintered reference materials in absolute values of the bending limit despite that fact that their density is significantly reduced by the filler addition.

The observed difference in failure modes of reference and syntactic foam materials can be linked to previous studies on fracture toughness of identical types of syntactic foams. Not surprisingly, these have indicated that failure tends to be more ductile in unfilled compared to syntactic foam materials, with a matching effect on crack propagation. Concerning crack propagation, some similarities can be found in other investigations concerning high dynamic testing documented in the literature: impact energies measured in Charpy impact tests performed on U- and V-notched samples proved to be one order of magnitude higher for the former type of specimen, irrespective of the shape of the notch. Similar differences were observed in J integral values [43].

The fact that case hardened reference samples exhibit a stepwise failure, may be interpreted as an expression of the change in material properties with increasing distance from the sample surface. This property gradient is of course the aim, and thus a logical consequence of case hardening, and as such, it is also reflected in the hardness measurements performed on the reference samples. It can be assumed that the drop in hardness towards the core reflected in Figure 5 and Figure 9 is correlated with a corresponding reduction in strength and a parallel increase in ductility. This may explain the observed crack arrest and the continuation of failure following an intermediary plastic phase as observed in some of these samples. The contrasting response of the case hardened syntactic foam samples confirms this understanding.

Altogether, these findings illustrate the very positive effect of the hardening treatments on the properties of syntactic iron foams which open new application areas. 

## 5. Conclusions

The present study has, for the first time, shed light on the susceptibility of iron matrix syntactic foams to thermo-chemical surface hardening treatments. The main questions addressed were whether the build-up of syntactic foam samples would significantly affect the heat treatment outcome, and whether the treatment itself would cause damage to the structure of the foam. The investigations have shown that treatments at parameter settings typical for non-porous materials, can in fact be transferred to syntactic foams. The depth of the case has been shown to be similar in both conventional and syntactic foam samples containing approximately 40 vol.% of hollow glass microspheres. Moreover, the increase in the bending limit obtained by the surface hardening treatments is similar for both groups of materials and reaches factors of approximately 3.5. Not surprisingly, this increase in strength coincides with a loss of ductility. The latter effect is more pronounced in the case of the syntactic foam samples, as here both the change of matrix properties caused by the heat treatment and the porosity adversely affects ductility.

In terms of the elastic modulus, a positive effect of heat treatment is observed for non-porous samples, indicating that in non-heat treated reference materials, plastic deformation is initiated already during the very early stages of deformation. In the case of syntactic foams, neither carburizing nor carbonitriding affects the elastic modulus in any statistically significant way. Surface hardened reference materials outperform syntactic foams in this respect once more by a factor of 2. However, the apparent advantage of the non-porous materials is somewhat alleviated if the reduced density (−34.5%) of the foams is taken into account, too, and weight-specific properties are compared.

In conclusion, it can be stated that thermo-chemical surface hardening treatments are a viable option for iron matrix syntactic foams, and can be performed in the same manner as would be the case for the unfilled matrix material. From a manufacturing technology and application point of view, this is an extremely important result, as it means that

- thermo-chemical treatments common for gears can be transferred to composite gears consisting of compact and porous, i.e., syntactic foam, components, and
- treatment parameters do not necessarily have to be adapted to accommodate special requirements of the syntactic foam components.

In effect, this means that in terms of secondary processes, composite gear wheels, which promise massive benefits in terms of weight reduction, vibration, and damping characteristics, can be treated just like their conventional counterparts without the need for additional dedicated process developments. This will greatly reduce any effort related to their introduction, and it will open up paths for the realization of other highly loaded engineering components on the basis of iron matrix syntactic foams.

Prior to this introduction, however, further research on material properties would be beneficial. This includes the quantification of vibration damping properties of syntactic foams in the as-sintered versus the surface hardened state, as well as the evaluation of these materials’ characteristics under cyclic loads to judge their performance under conditions of fatigue, which are important in gear wheel applications. The notion that effects specific to syntactic foams may be encountered in this respect, is based on the observation that for the material combination studied in the present case (hollow glass microspheres as filler), some effect of the thermal treatment on the structure of the spheres was observed. If this change turns out to have detrimental effects on, e.g., fatigue strength and consideration of alternative filler materials with increased temperature resistance might be of interest. 

According to the results of the presented investigations, syntactic foams represent an excellent basis for the application, for example, as filling material for lightweight gears. It can be assumed that the foams have a positive effect on the damping properties and will therefore be of particular future interest for gears that are running at high speeds, resulting in high excitation frequencies. It should be emphasized that the application of the current processing technology for carburizing and carbonitriding gears with the highest load capacity requirements is possible without adapting the heat treatment to the partial change in materials brought in by a syntactic foam insert.

In view of the alternative fields of application (e.g., decoupling support structures) besides the gear scenario, further studies with varied sphere contents in the range of 20–60 vol.% might be interesting and provide additional insights into the interaction of the materials’ 3D structure with the diffusing components during the hardening treatments. In view of the core application scenario, future studies should attempt to shed light on material properties under cyclic loads to provide additional data for dimensioning of components.

## Figures and Tables

**Table 1:** Properties of the used raw materials [33,34].

Material	Diafe 5100	S60HS
Chemical Composition (wt.%)	Fe > 97.7 C < 0.9 O < 0.5 N < 0.9	SiO_2_ appr. 70–80 Na_2_O appr. 3–8 CaO appr. 8–15 B_2_O_3_ appr. 2–6
Size Distribution	d(10) max. 1.2 μm d(50) max. 2.5 μm d(90) max. 3.5 μm	d(10) max. 12 μm d(50) max. 29 μm d(90) max. 48 μm
Other	Density 7.8 g/cm^3^ Sintering density (900 °C) 7.5 g/cm^3^	True density 0.6 g/cm^3^ Softening point 600 °C Crush strength 1240 bar

**Table 2:** Composition of mixtures of the test batches.

Masses	Batch 1	Batch 2
Diafe 5100 [g]	1884.00	1130.4
binder [g]	148.22	148.22
S60HS [g]	-	57.6

**Table 3:** Moulding parameters.

Parameter	Setting
Injection temperature	120 °C
Injection pressure	120 bar
Injection velocity	200 mm/s
Mould temperature	40 °C

**Table 4:** Results of the density measurements.

Material	Measured Density [g/cm^3^]	Comparison with Reference Value	
		Reference/Theoretical Value	Comparison
Sintered compact batch	7.09	7.787 (7.5)	91% (94.5%)
Sintered foam with S60HS spheres	4.65	4.96 (4.74)	94% (98%)

**Table 5:** Results of shrinkage measurements.

Directional Shrinkage	Compact Material	Syntactic Foam
Length shrinkage [%]	14.5	15.3
Width shrinkage [%]	14.2	15.2
Height shrinkage [%]	15.1	14.9

**Table 6:** Results of the S-OES measurements on compact samples in the as-sintered state (3 samples).

Element	C	P	S	Ni	Co	Ti
wt.%	0.007–0.02	<0.001	<0.001	<0.003	<0.006	<0.005

**Table 7:** Failure mechanisms under a 4-point bending load of reference samples without microspheres (compact material), and syntactic foam samples with and without case hardening treatment, as expressed in the force-deflection curves.

Heat Treatment	Compact Material	Syntactic Foam
Without	Elastic region followed by consecutive plastic deformation with a shift of the plastic zone from the loading span to in between the load introduction and support points. No fracture of the sample (Figure 11).	Elasto-plastic region followed by linear increase of the force deflection curve, indicating plastic deformation and subsequent progressive crack propagation until complete failure of the sample (Figure 11).
Case hardened	Elasto-plastic region followed by crack initiation, growth, arrest, and subsequent further plastic bending of the samples; in some cases, continued crack propagation after intermediate plastic deformation, the latter remaining within the loading span (Figure 12).	Elasto-plastic region followed by immediate, brittle failure of the sample (Figure 13).

## Data Availability

Data is contained within the article or Supplementary Materials.

## Supplementary Materials

The following are available online at https://www.mdpi.com/article/10.3390/ma14164358/s1, Figure S1: Example of a gear wheel with foam insert (Here: Al foam produced via the Foaminal^TM^ process). Inserted picture shows the added CFRP inlay required for sufficient transmission of torque due to the limited strength of the low density Al foam. The gear originates from a coopera-tion between Fraunhofer IFAM and WZL (RWTH Aachen). Figure S2: Overall process chain of the sample production, Figure S3: Injection mould for sample production, Figure S4: Time-temperature program for thermal debinding and sintering, Figure S5: Sample chamber of the bell furnace used for hardening treatments, Figure S6: Setup of the 4-point bending test.
