# Supplementary material for: Syntactic Iron Foams’ Properties Tailored by Means of Case Hardening via Carburizing or Carbonitriding
Source: Materials (Basel). 2021 Aug 4;14(16):4358. doi: 10.3390/ma14164358 (PMC8398341; doi:10.3390/ma14164358)
Supplement: Supplementary file 1 [file materials-14-04358-s001.zip › materials-1262969-supplementary.pdf]

materials

Supplementary Materials

Syntactic Iron Foams' Properties Tailored by Means of Case Hardening via Carburizing or Carbonitriding

Jörg Weise [1,*], Dirk Lehmhus [1], Jaqueline Sandfuchs [2,3], Matthias Steinbacher [2], Rainer Fechte-Heinen [2] and Matthias Busse [1]

[1] Department of Powder Technology, Fraunhofer Institute for Manufacturing Technology and Advanced Materials IFAM, Wiener Strasse 12, 28359 Bremen, Germany; dirk.lehmhus@ifam.fraunhofer.de (D.L.); matthias.busse@ifam.fraunhofer.de (M.B.)
[2] Department of Heat Treatment, Leibniz Institute for Materials Engineering IWT, Badgasteiner Str. 3, 28359 Bremen, Germany; ja.sandfuchs@gmx.de (J.S.); steinbacher@iwt-bremen.de (M.S.); fechte@iwt-bremen.de (R.F.-H.)
[3] Faculty of Production Engineering, University of Bremen, Bibliothekstraße 1, 28359 Bremen, Germany
* Correspondence: joerg.weise@ifam.fraunhofer.de

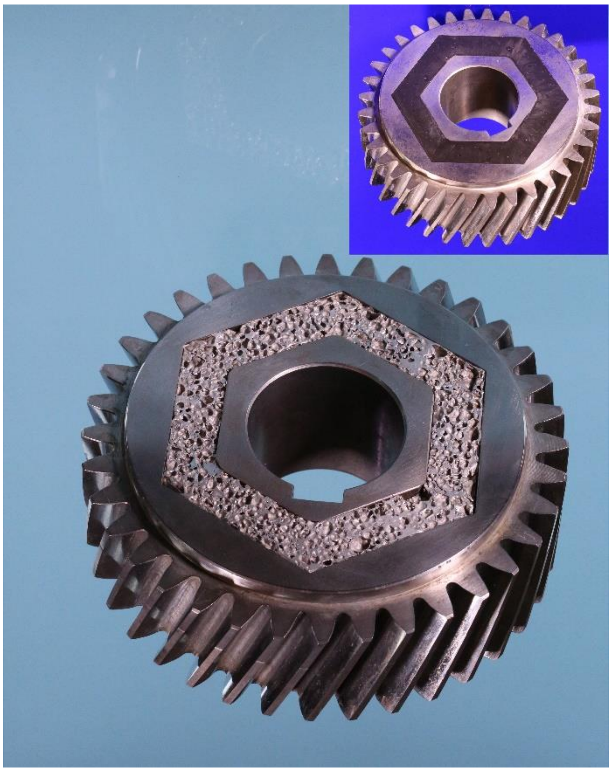

Figure S1. Example of a gear wheel with foam insert (Here: Al foam produced via the Foaminal™ process). Inserted picture shows the added CFRP inlay required for sufficient transmission of torque due to the limited strength of the low density Al foam. The gear originates from a cooperation between Fraunhofer IFAM and WZL (RWTH Aachen).

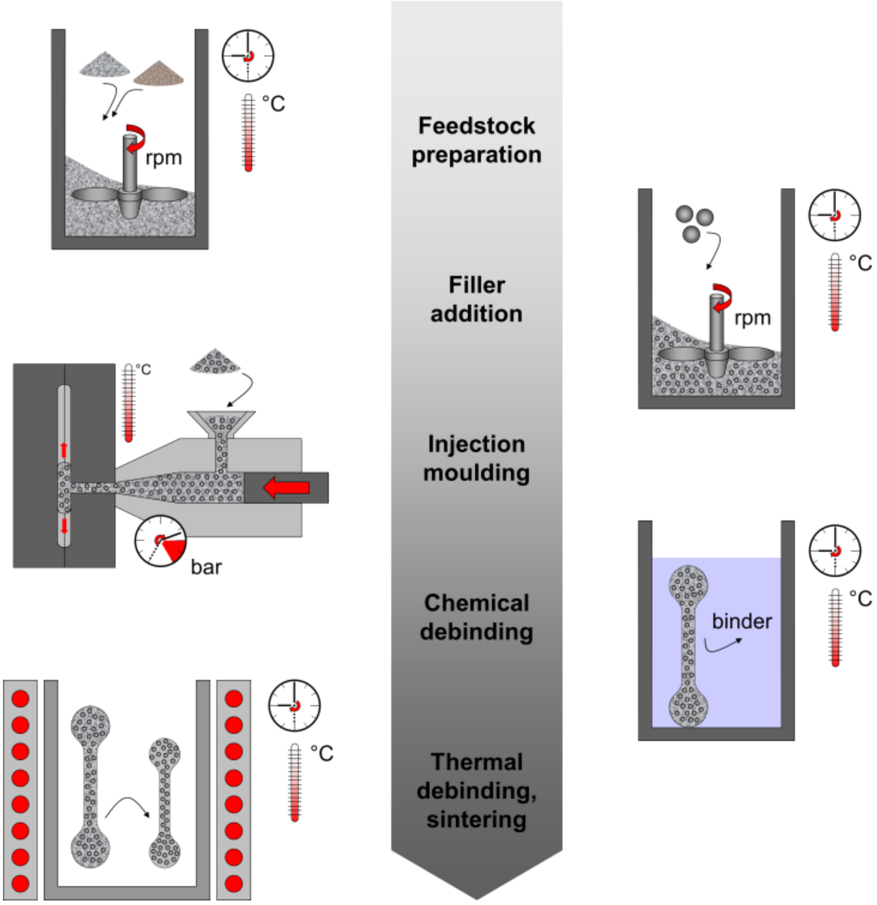

Figure S2. Overall process chain of the sample production.

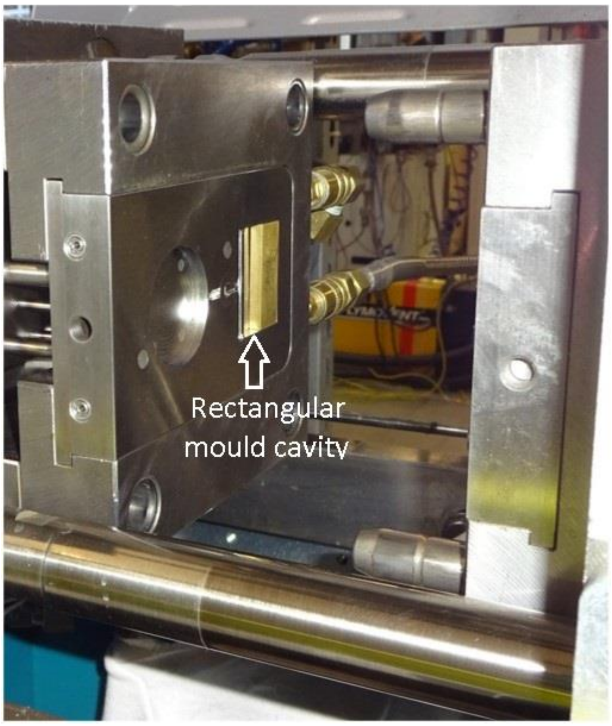

Figure S3. Injection mould for sample production.

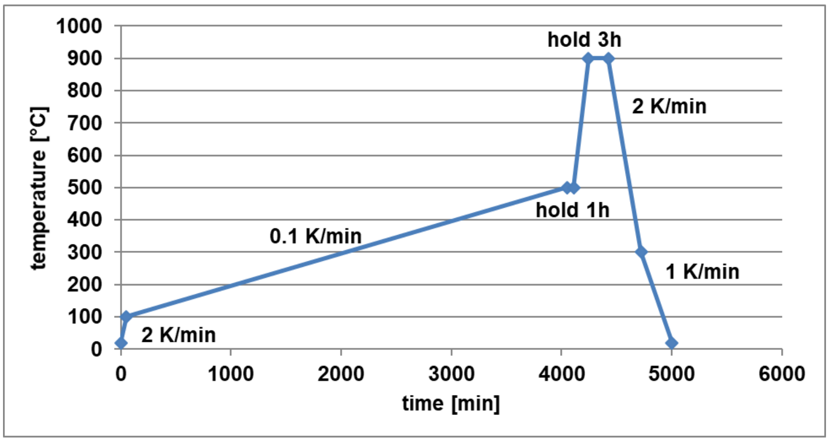

Figure S4. Time-temperature program for thermal debinding and sintering.

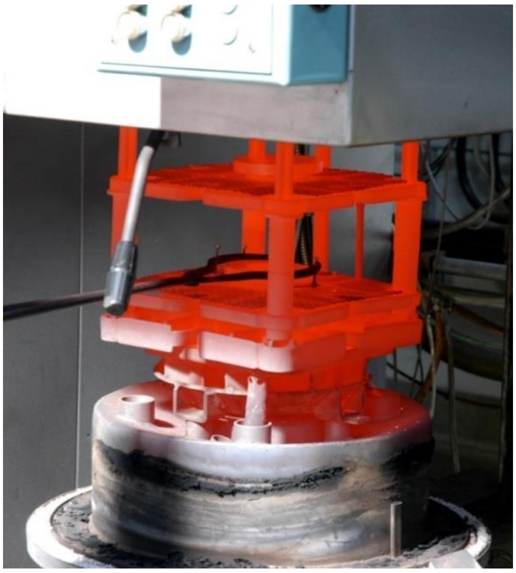

Figure S5. Sample chamber of the bell furnace used for hardening treatments.

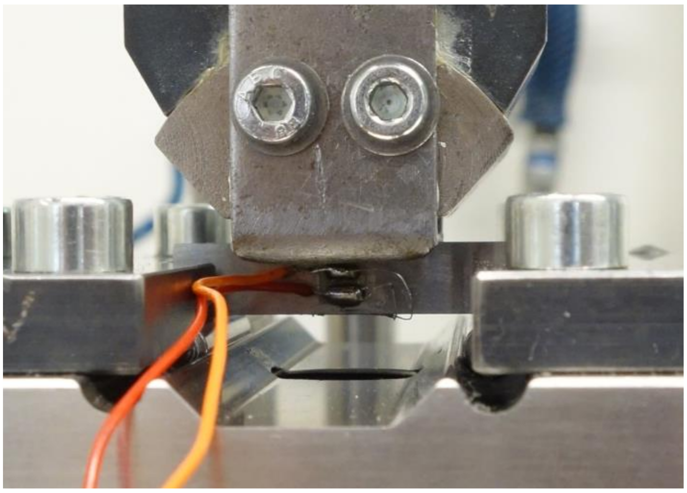

Figure S6. Setup of the 4-point bending test.
